# Feature level fine grained sentiment analysis using boosted long short-term memory with improvised local search whale optimization

**DOI:** 10.7717/peerj-cs.1336

**Published:** 2023-04-24

**Authors:** Lakshmi Revathi Krosuri, Rama Satish Aravapalli

**Affiliations:** Vellore Institute of Technology University, Guntur, Andhra Pradesh, India

**Keywords:** Sentiment analysis, Long short-term memory (LSTM), Levy flight-based mayfly optimization algorithm (LFMO), Whale optimization, Log term frequency-based modified inverse class frequency (LFMI)

## Abstract

**Background:**

In the modern era, Internet-based e-commerce world, consumers express their thoughts on the product or service through ranking and reviews. Sentiment analysis uncovers contextual inferences in user sentiment, assisting the commercial industry and end users in understanding the perception of the product or service. Variations in textual arrangement, complex logic, and sequence length are some of the challenges to accurately forecast the sentiment score of user reviews. Therefore, a novel improvised local search whale optimization improved long short-term memory (LSTM) for feature-level sentiment analysis of online product reviews is proposed in this study.

**Methods:**

The proposed feature-level sentiment analysis method includes ‘data collection’, ‘pre-processing’, ‘feature extraction’, ‘feature selection’, and finally ‘sentiment classification’. First, the product reviews given from different customers are acquired, and then the retrieved data is pre-processed. These pre-processed data go through a feature extraction procedure using a modified inverse class frequency algorithm (LFMI) based on log term frequency. Then the feature is selected *via* levy flight-based mayfly optimization algorithm (LFMO). At last, the selected data is transformed to the improvised local search whale optimization boosted long short-term memory (ILW-LSTM) model, which categorizes the sentiment of the customer reviews as ‘positive’, ‘negative’, ‘very positive’, ‘very negative’, and ‘neutral’. The ‘Prompt Cloud dataset’ is used for the performance study of the suggested classifiers. Our suggested ILW-LSTM model is put to the test using standard performance evaluation. The primary metrics used to assess our suggested model are ‘accuracy’, ‘recall’, ’precision’, and ‘F1-score’.

**Results and Conclusion:**

The proposed ILW-LSTM method provides an accuracy of 97%. In comparison to other leading algorithms, the outcome reveals that the ILW-LSTM model outperformed well in feature-level sentiment classification.

## Introduction

In recent times, the growth of web applications like ‘social networks’, ‘e-commerce websites’, ‘blogs’, and ‘online forums’ has allowed people to express their views on products, events, and services (Rosa et al., 2018). Similarly, sentences with positive connotations may indicate ‘happiness’, ‘contentment’, or ‘pleasure’. As a result, these data must be analyzed to extract pertinent data and polarized views, respectively. Furthermore, businesses and individuals must be able to determine whether a user’s opinion is beneficial or detrimental in real time. This reveals the significance of people’s opinions in society. Therefore, it must be formulated and expressed appropriately (Selvakumar & Lakshmanan, 2022; Sharma, Chaurasia & Srivastava, 2020). Sentiment analysis plays a vital role in identifying and converting people’s thoughts about a certain subject or item into a positive, negative, or neutral polarity or even into a score, or star rating (Sun et al., 2019). Based on the granularity preferred grade, sentiment analysis classification has been done at three levels that are ‘document level’, ‘phrase level’, and ‘feature or aspect level’.

The objective at the document level is to identify whether an entire opinion article shows a positive or negative attitude (Mutanov, Karyukin & Mamykova, 2021). Whereas in the phrase or sentence level, each statement is examined to determine whether it provided a positive, negative, or neutral attitude. On the aspect level, the finer-grained analysis is carried out and it investigates the viewpoint alone (Basiri et al., 2021). The assessments at the document and sentence levels do not specifically show what people liked and disliked. An opinion has two components that are an emotion which may be positive or negative and a target of opinion. The vast majority of sentiment analysis algorithms in use today concentrate on phrase- and document-level sentiment analysis (Ma et al., 2018). These two granularity-based sentiment analysis methods have partially overcome several issues, but they are still unable to satisfy the needs of the vast majority of current applications. Because of this, there has been a lot of interest in the expansion of fine-grained aspect-level sentiment analysis algorithms in the field of vision (Sadr, Pedram & Teshnehlab, 2019). Based on the methodology used, sentiment analysis at the aspect level may be further divided into lexicon-based, features-based, unsupervised, and more recently, deep learning-based categories (Kumar et al., 2020). Natural language processing (NLP) is the actual processing of text components. It converted the text element into a machine-readable format. The fundamental intention of NLP is sentiment analysis, which has several applications in ‘web mining’, ‘text mining’, and ‘data mining’ (Abdi et al., 2019). The quantity of false positives in text categorization is decreased by the application of deep learning algorithms (Souma, Vodenska & Aoyama, 2019; Wang, Niu & Yu, 2019). These deep learning methods have lately been used for several NLP problems for the categorization of small text (Zhai et al., 2020). However, identifying the proper situations for particular qualities is the key challenge. The majority of earlier methods that merged attentional processes with recurrent neural networks eventually increased noise and decreased prediction accuracy. Another challenge for attention systems is that the mood of some context words varies based on a variety of variables and cannot be inferred only from their appearance (Yang et al., 2020; Rehman et al., 2019). The remaining sections of the work carried are organized as follows:

Section 2 contains the literature survey. The research gap is described in Section 2.1, and the research methodology is discussed in Section 3. The final section discusses the probable outcomes and assessment metrics involved in this study.

## Literature survey

This section is a detailed evaluation of the research work done previously on the topic of sentiment analysis.

Numerous e-commerce and social media platforms allow customers to write large numbers of product evaluations online, which provides developers with invaluable information for building new items. Therefore, Vijayaragavan, Ponnusamy & Aramudhan (2020) have utilized a cluster-based classification method for online product evaluations. Here, a support vector machine (SVM) classification method was employed to categorize the online customer product reviews. The second stage involves extracting the traits using an emotional analysis strategy. Finally, the effectiveness of the customer’s capacity to successfully buy the items is evaluated using fuzzy-based soft set theory. On the other hand, Wang & Wan (2019) have offered SentiGAN and C-SentiGAN as tools for obtaining emotional intelligence. This model includes a multi-class discriminator and various generators. Here, a penalty-based aim was employed to motivate generators to produce a range of instances for a particular mood label. Similarly, Ishaq, Asghar & Gillani (2020) have presented a genetic algorithm with a convolutional neural network to provide a sentiment analysis classification method. Here the feelings were examined by combining three processes, such as mining semantic characteristics, utilizing Word2vec to transform the retrieved *corpus*, and deployment of CNN for opinion mining. Likewise, Shuang et al. (2020) have established a feature distillation network (FDN) for minimizing irrelevant data (noise) and extracting feature-related emotional information. The relationships among aspects and their respective contexts are implemented at a high resolution using a unique double gate technique. Xu et al. (2019) have presented an improved word representation strategy that builds weighted word vectors using the well-known TF-IDF algorithm and sentiment analysis. Bidirectional long short-term memory (BiLSTM) receives the weighted word vectors and correctly collects context information, improving the representation of comment vectors.

On the other hand, Ikram & Afzal (2019) have introduced aspect-based sentiment classification to detect hidden patterns in academic huge data by recognizing aspect-level sentiments. Similarly, Ye, Xu & Luo (2021) have presented an ALBERTC-CNN based aspect level sentiment analysis. The upgraded ALBERTC network was utilized to extract global phrase information and local emotion data while representing the initial aspect-level text as a word vector. Additionally, Gao et al. (2019) have suggested the CE-HEAT approach to extract the uncommon sentiment terms. It has two hierarchical attention units, the first one collects sentiment characteristics from the sentiment attention layer. The alternative one makes use of the aspect characteristics obtained *via* the aspect attention layer.

Boussaïd, Lepagnot & Siarry (2013) provided a survey about a few of the key metaheuristics. It describes the components and concepts used in numerous metaheuristics in order to compare and contrast their similar and distinct characteristics. Similarly, Dokeroglu et al. (2019) have distinguished fourteen novel and phenomenal metaheuristics that have been invented in the last 20 years, in addition to the classic ones such as genetic, tabu search and particle swarm. Their study addresses critical metaheuristic issues as well as new recommendations for potential research opportunities and open challenges of nature-inspired population-based optimization algorithms. Hussain et al. (2019) conducted a survey of metaheuristic research in the literature, which included 1,222 publications from 1983 to 2016. Based on the evidence gathered, their article explored four aspects of metaheuristic research: the introduction of new algorithms, comparisons and analysis, modifications and hybrids, future directions and research gaps. Baydogan & Alatas (2021) proposed an automatic hate speech detection system based on metaheuristic approach for achieving better results in their suggested methodology. Ant lion optimization (ALO) and moth flame optimization (MFO) algorithms were developed for the hate speech detection problem in the suggested optimization technique. Baydogan (2021) have presented a novel swarm intelligence-based social spider algorithm (SSA), which is primarily focused on a modeling of spider collaborative behaviours and was initially used for sentiment analysis (SA) on Twitter data., introducing a new application domain for optimization algorithms.

### Research gap

- The domain dependence on sentiment terms is the major obstacle to opinion mining and sentiment analysis. A feature set may perform exceptionally well in one domain while exhibiting very poor performance in another.
- For sentiment analysis to be effective, opinion words must interact with implicit data. The implicit information determines how sentimental phrases actually work.
- People express their ideas in many ways. Every person has a unique opinion since everyone has a distinct manner of thinking and expressing themselves.
- Typographical flaws can occasionally make it difficult to gather opinions.
- Natural language overhead, such as ambiguity, co-reference, implicitness, inference, *etc*., made sentiment analysis tools more difficult to use.
- It might be difficult to classify sentences as positive or negative, very positive, very negative, and neutral in terms of opinion mining (OM) at the sentence level since every writer has a distinct writing style and because a sentence may include positive or negative, very positive, very negative, and neutral concepts.

## Research methodology

Reviews, blogs, news articles, and comments are just a few methods by which people express their opinions. Sentiment analysis is employed to swiftly extract insights from vast amounts of textual data, thereby boosting service quality and generating enormous profits for enterprises.

- Data collection
- Data pre-processing—tokenization, lemmatization, stemming, removal of stop words.
- Feature extraction—log term frequency-based modified inverse class frequency (LFMI).
- Feature selection—levy flight-dependent mayfly optimization algorithm (LFMO).
- Sentiment classification—improvised local search whale optimization boosted long short-term memory (ILW-LSTM).

### Proposed methodology

**Figure 1:**
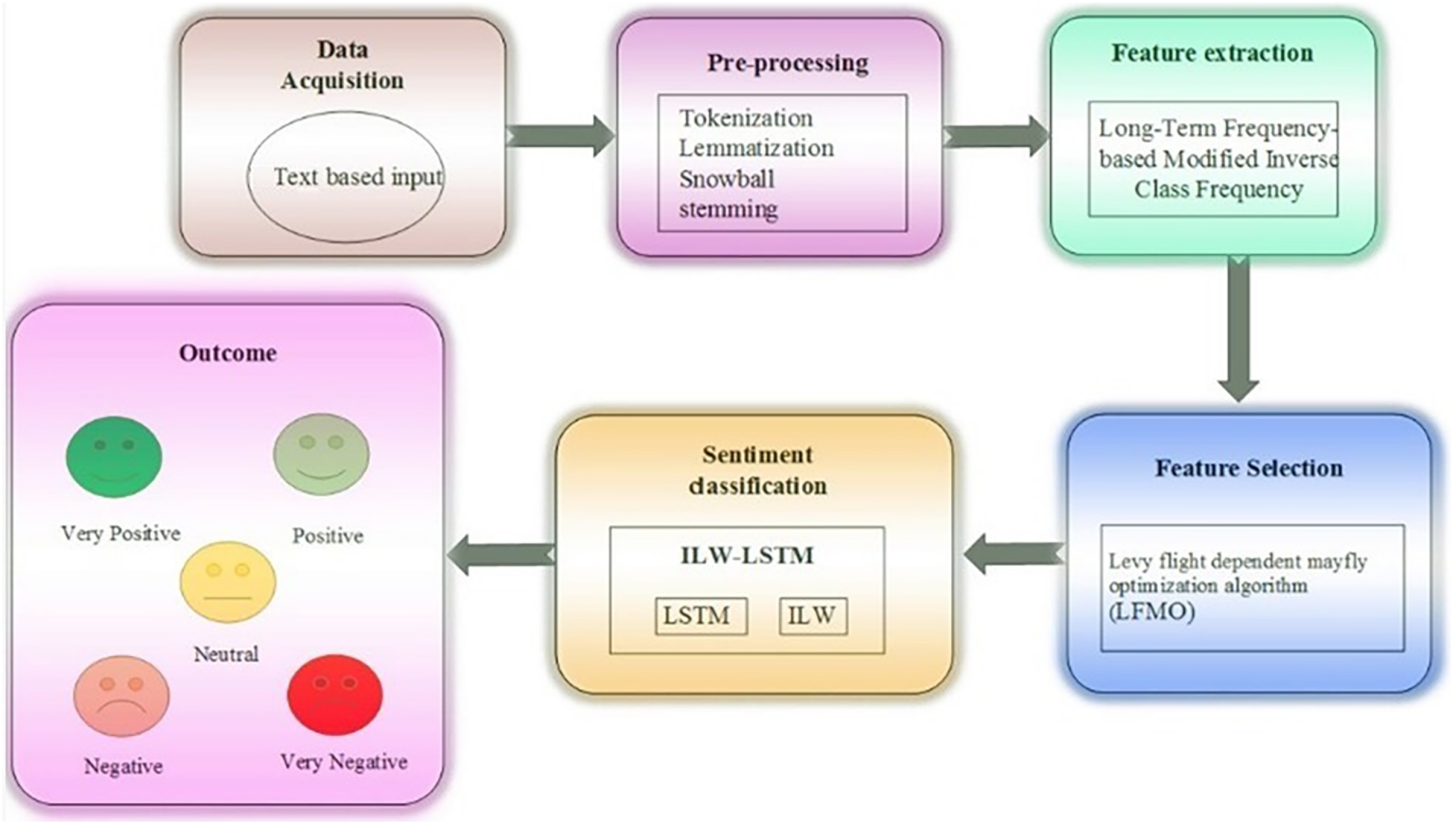
Proposed framework.

In this article, ILW-LSTM, a deep learning approach, is suggested for the feature-level sentiment analysis. This method includes five main stages such as ‘data acquisition’, ‘data pre-processing’, ‘feature extraction’, ‘feature selection’, last ‘sentiment classification’. At first, the data is gathered from the taken dataset then the textual data is taken for sentiment analysis to determine the essential aspects. The textual data is subjected to pre-processing, which includes ‘white tokenization’, ‘lemmatization’, and ‘snowball stemming’. Tokenization is a technique for dividing text into smaller bits. Text in huge amounts is broken down into words or phrases. Depending on the issue, precise data criteria are defined to divide the text content into pertinent tokens. One of the most used data pre-processing techniques is lemmatization. The suggested method makes use of the long-term frequency-based modified inverse class frequency for textual feature extraction. Subsequently, the Levy flight-dependent mayfly optimization algorithm (LFMO) is employed to improve the classification accuracy. This method subjects to the training model and chooses the optimal set of features. The final step is to add the selected characteristics to the proposed ILW-LSTM for polarity classification. The output of the ILW-LSTM represents the classes as ‘positive’, ‘negative’, ‘very positive’, ‘very negative’, and ‘neutral’ (sentiments) of the entered data. Figure 1 displays the general stream diagram of the suggested feature-level sentiment analysis.

### Data acquisition

A rating is created by combining information from the review about the sentence subjects and attitudes. Below are the pre-processing steps for the customer reviews that will be processed further.

### Pre-processing

Pre-processing procedures are employed to remove undesirable datasets from the group. In this case, pre-processing is carried out as part of the sentiment analysis data preparation procedure. The three pre-processing operations are ‘white space tokenization’ ‘lemmatization’, and ‘snowball stemming’.

#### Tokenization

To understand the context or build the NLP model, this method separates text into words or tokens whenever it encounters a whitespace character. To determine the meaning of the text, it further examines the word order.

#### Lemmatization

The process of lemmatization uses morphological analysis and vocabulary to determine the lemma. It also yields the dictionary structure of the word. Morphological analysis is the process used to identify all possible connections in a multidimensional collection. It is also employed for multidimensional issue-solving. It transforms lemma into something akin to a byte sequence so you may create a duplicate of your preferred lemmatize. The relationship between the term’s regular form and one of these words in a phrase is known as a lexeme. Additionally, part-of-speech (POS) labeling aims to improve the review's consistency by focusing on recall and accuracy. To the extent possible, this may be applied to the lexicons under investigation to eliminate groupings of terms judged unnecessary or disruptive to document identification.

#### Snow-ball stemming

Stemming is a NLP technique that reduces word inflection to its root forms, when words of a similar kind clump together under a single stem. The SBS, also known as the Porter2 stemming algorithm since it is a superior adaption of the Porter Stemmer, is utilized in this situation. By changing the context of a word ending sequence in a probabilistic manner, it transforms terms into stems. The stemming procedure is one that historically ignored the word’s few characters without considering its significance. Lemmatization, however, is a technique that changes words into meaning without eliminating any characters.

### Feature extraction

The method of feature extraction is crucial to sentiment analysis. It is mainly employed to convert raw or original data into a low-dimensional feature. The characteristics from the text are taken out in this part for sentiment analysis. Our suggested method uses a log-term frequency-based modified inverse class frequency (LFMI) for further evaluation. Additionally, this method incorporates testing and training, with the extraction of textual elements occurring during training.

#### Log term frequency-based modified inverse class frequency

The word usually weighting of the input reviews is carried out after the pre-processing operation. The frequency of a word in a document of review sentences is measured by the term frequency $\; {T_{fq}}$ Zhao et al. (2021). However, ${T_{fq}}$ alone is insufficient since a text will be heavily influenced by the terms that appear more frequently. The use of the class information from reviews in supervised term weighting techniques has drawn increasing interest. ${T_{fq}}\;$ and a supervised term weighting method are thus combined in this case. Inverses class frequency ( ${I_{fq}}$) is the proportion of total classes to total classes where the phrase appears on training reviews. Before performing log normalization on the final output of ${T_{fq}}$ data, which is denoted as long term frequency or $\; L{T_{fq}}$. LTF-centered term weighting is computes ${T_{fq}}$ of each term in the pre-processed dataset. The modified form of $\; M{I_{fq}}$, known as the MICF, is then computed for each word. In this case, the change of $M{I_{fq}}$ is carried out because distinct class-specific scores for each term must have varying contributions to the overall term score. As a result, it is necessary to apply various weights to the various class-specific scores, and the weighted total of all the class-specific scores is then used as the final term ‘score’. The suggested calculation for term weighting using the aforementioned concoction of schemes is written as Eq. (1)

(1) $$L{T_{fq}} - M{I_{fq}}\left( {{t_m}} \right) = L{T_{fq}}\left( {{t_m}} \right)*\mathop \sum \nolimits_{n = 1}^x {w_{mn}}.\left[ {{I_{fq}}\left( {{t_m}} \right)} \right]$$

where ${w_{mn}}$ stands for the particular weighting factor for the term ${t_m}$ for class ${c_m}$, which is well-defined as

(2) $${w_{mn}} = \log \left( {1 + {{{r_x}\mathord{\buildrel{\lower3pt\hbox{$\scriptscriptstyle\rightharpoonup$}}\over  t} } \over {max\left( {1,{r_x}\mathord{\buildrel{\lower3pt\hbox{$\scriptscriptstyle\leftarrow$}}\over  t} } \right)}}.{{{r_x}\tilde t} \over {max\left( {1,{r_x}\hat t} \right)}}} \right)$$

The procedure used to assign weight to the available datasets is known as the weighting factor. Where ${r_x}\mathord{\buildrel{\lower3pt\hbox{$\scriptscriptstyle\rightharpoonup$}}\over  t}$ denotes the number of reviews ${r_x}$ in class ${c_m}$ that contain the term $\; \; {t_m}$, ${r_x}\mathord{\buildrel{\lower3pt\hbox{$\scriptscriptstyle\leftarrow$}}\over t}$ denotes the number of ${r_x}$ in other classes that contain the term $\; {t_m}$. $\; {r_x}\widetilde {t\; }\;$denotes the number of ${r_x}$ in other classes that do not contain the term ${t_m}$, and ${r_x}\hat t$ denotes the number of reviews ${r_x}$ in class ${c_m}$ that do not contain the term $\; {t_m}$. Negative weights are disregarded using the constant 1. To prevent the zero-denominator problem in the worst-case scenario, the minimal denominator is set to 1 if ${r_x}\hat t = 0$ or ${r_x}\mathord{\buildrel{\lower3pt\hbox{$\scriptscriptstyle\leftarrow$}}\over t}$. The MICF serves as the focal point of a new term weighting created under the name $L{T_{fq}} - M{I_{fq}}\left( {{t_m}} \right)$. The $L{T_{fq}}\left( {{t_m}} \right)$ and ${I_{fq}}\left( {{t_m}} \right)$ formulas may be written as

(3) $$L{T_{fq}}\left( {{t_m}} \right) = \log \left( {1 + {T_{fq}}\left( {{t_m},{r_x}} \right)} \right)$$

where ${T_{fq}}\left( {{t_m},{r_x}} \right)$ represents the total frequency of a phrase ${t_m}$ occurring on the set of documents $\; rev{r_x}$.

(4) $${I_{fq}}\left( {{t_m}} \right) = \log \left( {1 + \displaystyle{N \over {C\left( {{t_m}} \right)}}} \right)$$

where $C\left( {{t_m}} \right)$ is the number of classes that include the word ${t_m}$, and $N$ denotes the total number of classes in a set of document reviews. ${F_x} = \; {F_1},\; {F_2},......{F_3},.....{F_p}$ after term weighting denotes the features of the dataset, where ${F_1},\; {F_2},......{F_3},.....{F_p}$ denotes the number of weighted terms from the pre-processed dataset.

### LFMO-based feature selection

The study implements feature selection using the optimization technique known as LFMO, which is described as,

The mayfly algorithm was inspired by the way that mayflies interact with one another, particularly during mating. Once the eggs hatch, then the mayflies are immediately regarded as adults. Despite how long they are alive, only the fittest mayflies tend to survive. Every mayfly in the search space has a position that correlates to a method for resolving the problem. The traditional mayfly method uses RANDOM functions to generate new variables that result in the local optimum. Here, Levy flying and the Mayfly algorithm were coupled by the researchers to increase the mayfly’s ability to seek and determine the best solution. According to the Levy flight notion, if a Levy flight-based technique is used for structural analysis, it provides quick convergence rate and does not require any derivative information. It also improves the local search avoidance and local trapping of the ideal solution. The following stages are necessary for the proposed mayfly optimization method to function:

***Stage 1*:** There should be two sets of mayflies, one for the male population and the other for the female. Then, each and every mayfly is randomly positioned in the problem space as a candidate solution, indicated by the d-dimensional vector ${Q_{Gx}} = \left( {{Q_{G1}}, {Q_{G2}} , \ldots ,{Q_{Gd}}} \right).$ Then, depending on the established goal function $F\left( {{C_T}\left( {{Q_{Gx}}} \right)} \right)$, the performance is evaluated (Nagarajan et al., 2022; Zervoudakis & Tsafarakis, 2020).

***Stage 2*:** The initialization of a mayfly’s velocity $vel = \left( {ve{l_1}, \ldots ,ve{l_d}} \right)$ occurs during a positional change. Hybrid interplay between individuals and social flying experiences determines its path. Every mayfly adapts to change its route to match its current personal optimal position $\left( {Pbest} \right)$. Additionally, it also changes dependent on the best position that any other mayfly in the swarm has obtained thus far $\left( {Gbest} \right)$.

***Stage 3*:** With initialized velocities of $ve{l_{mx}}$, the population of male mayflies is designated as ${Q_{Gmx}}\left( {x = 1,2, \ldots ,\; IG} \right)$. The male population mayflies, which congregate in swarms, indicate that each mayfly’s position changes on its personal experience and that of its neighbors. $Q_{Gx}^T$ is taken to be mayfly *x*’s current location in search space at time step *T*, and it changes as velocity $vel_x^{T + 1}$ is added to the current position. This expression is written exactly as it is provided here.

(5) $$Q_{Gmx}^{T + 1} = Q_{Gx}^T + vel_x^{T + 1}$$

A few meters above the water, with $Q_{Gxm}^0U\left( {{Q_{Gmmin}}, {Q_{Gmmax}}} \right)$, male mayflies are thought to be present and engaged in a nuptial dance. Since they are always moving, it may be inferred that these mayflies don’t have remarkable speeds. The velocity *x* of a male mayfly is therefore determined as follows.

(6) $$vel_{xy}^{T + 1} = g*vel_{xy}^T + {m_1}{e^{ - \beta r_p^2}}\left( {Pbes{t_{xy}} - Q_{Gmxy}^T} \right) + {m_2}{e^{ - \beta r_g^2}}\left( {Gbes{t_y} - Q_{Gmxy}^T} \right)$$

Here, the contributions of the social and cognitive components are scaled up using positive attraction constants $\; \; {m_1}$. $vel_{xy}^T\; \;$ relates to the mayfly *x*’s velocity in dimension $y = 1, \ldots , i$ at time step *T*. $Q_{Gmxy}^T$ specifies the mayfly’s ${x^{th}}$ position in dimension j at time step $T,{m_1}$. Likewise, ${r_g}$ stands for the Cartesian distance between ${Q_{Gx}}$ and $Gbest$, whereas ${r_p}$ represents the distance between ${Q_{Gx}}$ and $Pbes{t_x}$. Additionally, $Pbes{t_x}$ designates the mayfly in its finest location yet. The personal optimal position $Pbes{t_{xy}}$ at the following time step $T\; + \; 1$ is calculated based on the minimization issues under consideration.

(7) $$Pbes{t_x} = \left\{ {\matrix{ {Q_{Gmx}^{T + 1},\; if\ f\left( {Q_{Gmx}^{T + 1}} \right) < F\left( {Pbes{t_x}} \right)} \cr {remains\; the\; same,\; \; \; \; otherwise} \cr } } \right.$$

The formula for the $Gbest$ position at step *T* time is given below.

(8) $$\eqalign {Gbest &\in \left\{ {Pbes{t_1},\; Pbes{t_2}, \ldots ,\; Pbes{t_I},\left| {F\left( {Cbest} \right)} \right.} \right\} \cr&= \min \left\{ {F\left( {Cbes{t_1}} \right),\; F\left( {Cbes{t_2}} \right), \ldots ,\; F\left( {Pbes{t_{IG}}} \right)} \right\}}$$

Here is the formula for calculating the Cartesian distance.

(9) $${\parallel}{{Q_{Gmx}} - {X_x}}{\parallel} = \sqrt {\mathop \sum \nolimits_{y = 1}^i {{\left( {{Q_{Gmxy}} - {X_{xy}}} \right)}^2}}$$

For the algorithm to work as intended, the finest mayflies in the swarm must repeatedly execute the up-and-down nuptial dance. Therefore, it is necessary to continuously adjust the velocity of these best mayflies, which is computed as follows.

(10) $$vel_{xy}^{T + 1} = vel_{xy}^T + \; d \times b$$

Here, d stands for the coefficient of nuptial dance, and b is the random rate between [1, 1].

***Stage 4*:** The population of female mayflies is initialized ${Q_{Gfx}}\left( {x = 1,2, \ldots ,IG} \right)$ with velocities $ve{l_{fx}}$ in this stage. Female mayflies often do not swarm as males do. Instead, it usually flies in the direction of its male peers to mate. $Q_{Gfx}^T$ is taken to be the location of female mayfly *x* in search space at step T time, and its position changes as a result of adding velocity $vel_x^{T + 1\; }\;$ to the current position.

(11) $$Q_{Gfx}^{T + 1} = Q_{Gfx}^T + vel_x^{T + 1}$$

$Q_{Gxm\; }^0U\left( {{Q_{Gmmin}},{Q_{Gmmax}}} \right)$ prevents one from randomizing the attraction process in this case. Thus, it is agreed that the model would be a deterministic process. Since there are issues with minimization, their velocities are calculated as given below.

(12) $$vel_{xy}^{T + 1} = \left\{ {\matrix{ {g*vel_{xy}^T + {m_2}{e^{ - \beta r_{mf}^2}}\left( {Q_{Gmxy}^T - Q_{Gfxy}^T} \right)\; if\; F\left( {{Q_{Gfx}}} \right) > F\left( {{Q_{Gmx}}} \right)} \cr {g*vel_{xy}^T + Fl \times b,\; if\; F\left( {{Q_{Gfx}}} \right) \le F\left( {{Q_{Gmx}}} \right)} \cr } } \right.$$

Here, $b$ stands for the random value in the interval [1, 1], and $Fl$ stands for the random walk coefficient. Equation (15) is used to get this value.

***Stage 5*:** A mayfly candidate solution’s velocity is determined in this stage using the Levy flight method. To determine the mayfly candidate’s speed, Eq. (13) is utilized.

(13) $$vel_{xy}^{T + 1} = \left\{ {\matrix{ {ve{l_{max}},\; if\quad vel_{xy}^{T + 1} > ve{l_{max}}} \cr { - ve{l_{max}},\; if\quad vel_{xy}^{T + 1} < - ve{l_{max}}} \cr } } \right.$$

In this step, the global finest component’s location is changed using the Levy flight method. Although the Levy flying approach has so far been applied for exploration, it is connected to a particular search. Here, $ve{l_{max}}$ is determined using the formula:

(14) $$ve{l_{max}} = levy\left( \lambda \right)*\; \left( {{Q_{Gmmax}} - \; {Q_{Gmmin}}} \right)$$

were, $levy(\lambda ) = 0.01{{{r_5}^\sigma } \over {{{\left| {{r_6}} \right|}^{1/\beta }}}}.$. $levy\left( \lambda \right)$ specifies the step length and incorporates the infinite variance of the Levy distribution and mean values of $1 < \; \lambda \; < 3$. $\lambda$ represents the step length. The distribution factor is indicated by the symbol.

***Stage 6:*** The gravitational constant, or $g$ value, can be thought of as a stable integer between $\left( 0 \right.,\left. 1 \right]$.

(15) $$g = {g_{max}} - \displaystyle{{{g_{max}} - {g_{min}}} \over {iteratio{n_{max}}}} \times Iteration$$

where $\; Iteration$ represents the algorithm’s current iteration, $iteratio{n_{max}}$ denotes the max number of iterations, and ${g_{max}},{g_{min}}$ signify the max and min values that may be considered for the gravity coefficient, respectively.

***Stage 7*:** Mayflies mate and the young are inspected. The crossover operator described here discusses the mating behavior of the mayflies. Each parent is chosen from the male and female population using the same selection method, which is the attractiveness of females to men. One might choose the parents specifically based on fitness function or at random. In terms of the fitness function, the best female mates with the best male, the second-best female mates with the second-best male, *etc*. This crossing produces two offspring, for which the following formulation is provided.

(16) $$offspring1 = L \times male + \left( {1 - L} \right) \times female$$

(17) $$offspring2 = L \times female + \left( {1 - L} \right) \times male$$

**Figure 2:**
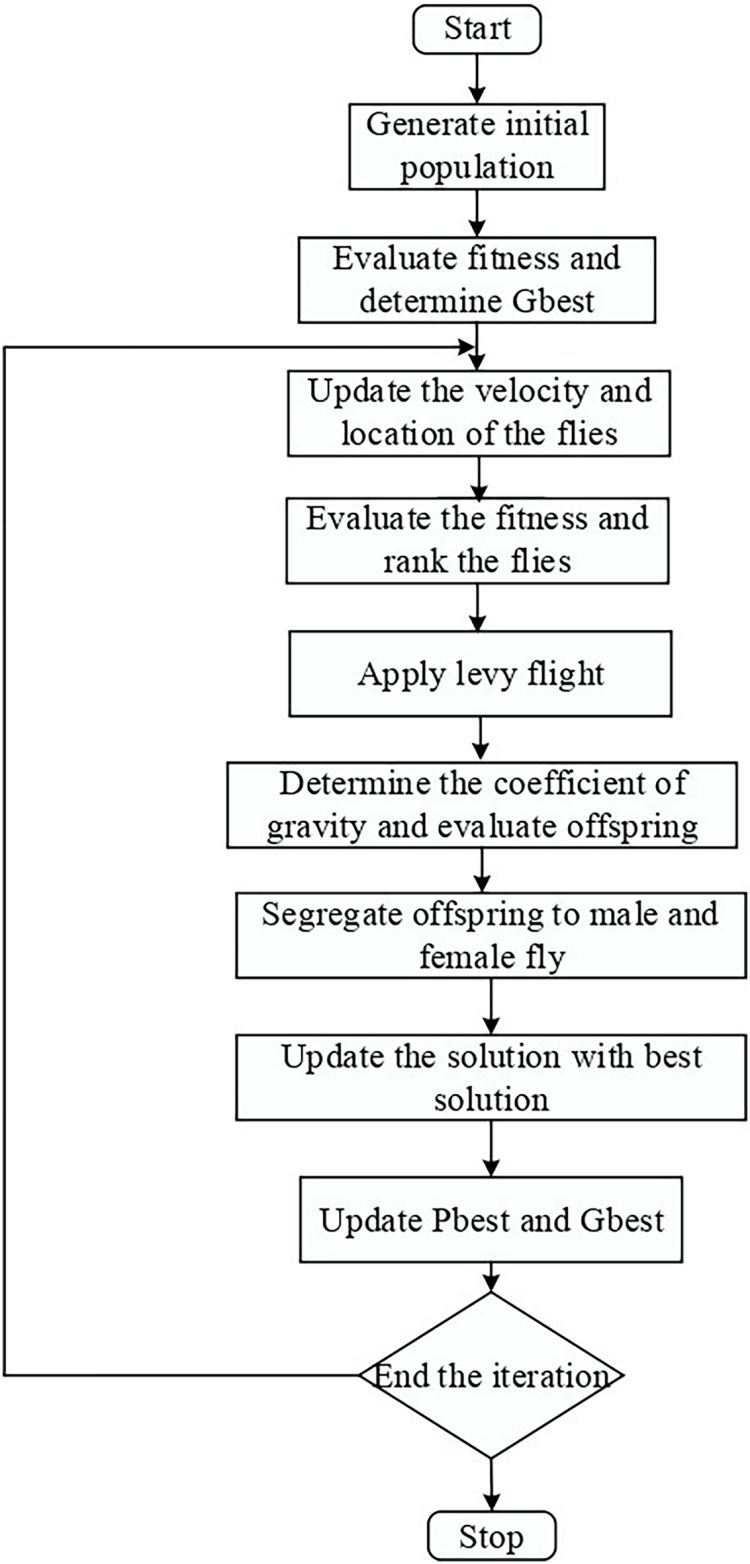
LFMO flowchart.

Here, the male stands for the male parent, the female for the female parent, and L is a random value falling inside a certain range. The offspring’s starting velocity is set at zero. Finally, a new subset of articles with additional educational elements is the outcome of this step. Figure 2 depicts the flow chart for the LFMO-based feature selection algorithm.

### Sentiment classification

The selected features from the feature selection process are inputted into the suggested ILW-LSTM classifier, to classify sentiment. A unique variety of RNNs called LSTM was created to address the vanishing and exploding gradients problem that recurrent neural networks encounter. Like other RNN types, LSTMs produce their output based on the data from the current time step and the output from the previous time step and then transmit their current output to the subsequent time step. A memory cell that can preserve its state for any length of time makes up each LSTM unit, as well as three non-linear gates: an input gate $\left( {i{n_t}} \right)$ a forget gate $\left( {f{g_t}} \right)$, and an output gate $\left( {ou{t_t}} \right)$. Information entering and leaving the memory cell is managed by these gates. Assume $tanh\left( . \right)$ as hyperbolic tangent function, $\odot$ as dot product, and $\sigma \left( . \right)$ as a sigmoid function applied to elements. At time t, $i{n_t}\; and\; hi{d_t}$ represent the input and hidden state vectors, respectively. Gate weight matrices are displayed by X and Y, while bias vectors are denoted by bias. By producing a number in the range [0, 1], the forget gate determines what data has to be forgotten.

**Algorithm:** ILW.

Initialize the population as ${{D}_{i}}$, where *i* = 1, 2, …, *ne*.
Evaluate each search agent’s fitness value.
The best search agent is ${{D}^{*}}$.
The maximum number of iterations is indicated by ${ite}{{r}_{{max}}}$.
While ( ${iter} \lt {ite}{{r}_{{max}}}$)
For each search agent
The value of *b*, *P*, *Q*, *r*, and *I* are updated
If ${f}\left( {{i} - 1} \right) \gt f\left({i} \right)$
Evaluate *r* using Eq. (31)
Else
Evaluate *r* using Eq. (28)
End if
If ${r} \lt 0.5$
If $\left| {W} \right| < 1$
Update the solution by Eq. (17)
Else if $\left| {W} \right| \ge 1$
Select ${{D}_{{randam}}}$
Equation (31) is used to update the solution
End if
Else if ${r} \ge 0.5$
Equation (28) is used to update the solution
End if
End for
Analyze the fitness of each search agent.
If better obtain an update ${{D}^{*}}$
Iter = iter+1
End while
Return ${{D}^{*}}$

(18) $$f{g_t} = \sigma \left( {{Y_{fg}}hi{d_{t - 1}} + {X_{fg}}i{n_t} + bia{s_{fg}}} \right)$$

The following equations are used by the input gate to compute $i{n_t}$ and ${\tilde c_t}$, combine them, and decide what additional information should be stored.

(19) $$i{n_t} = \sigma \left( {{Y_{in}}hi{d_{t - 1}} + {X_{in}}i{n_t} + bia{s_{in}}} \right)$$

(20) $${\tilde c_t} = tanh\left( {{Y_c}hi{d_{t - 1}} + {X_c}i{n_t} + bia{s_c}} \right)$$

(21) $${c_t} = f{g_t} \odot {c_{t - 1}} + i{n_t} \odot {\tilde c_t}$$

The following equations determine which components of the cell state should be output by the output gate.

(22) $$ou{t_t} = \sigma \left( {{Y_{out}}hi{d_{t - 1}} + {X_{out}}i{n_t} + bia{s_{out}}} \right)$$

(23) $$hi{d_t} = ou{t_t} \odot tanh\left( {{c_t}} \right)$$

The optimization of the LSTM using ILW is described as follows.

#### Optimization of LSTM based on ILW

The foundation for WOA is the humpback whales’ hunting method. The biggest member of the baleen whale family is the humpback whale. The unique hunting technique used by humpback whales is one of its uncommon features. These whales can recognize the victim and surround them. Equations (24) and (25) illustrate how the encircling movement is expressed

(24) $$W = \left| {Q.{D^*}\left( i \right) - D\left( i \right)} \right|$$

(25) $$D\left( {i + 1} \right) = {D^*}\left( i \right) - P.W$$

Here, P and Q are the vectors of the coefficient, ‘.’ designates the element-by-element multiplication, and D is the coordinates that detail the best outcome that was attained. The coefficient vectors P and Q are calculated *via* the following equation.

(26) $$P = 2b.randv - b$$

(27) $$Q = 2.randv$$

where b is exactly decreased across all iterations from 2 to 0, and $randv$ is a random vector between [0, 1]. There are two ways to mathematically express the humpback whale bubble-net approach; the decreasing encircling mechanism lowers the value of b in Eq. (26). The first assessment made by the spiral updating position strategy is the separation between the prey’s position at $\left( {{i^*}, {j^*}} \right)$ and the whale’s position at $\left( {i, j} \right)$. The spiral equation connecting the position of the whale and its prey is then calculated to replicate the helix-shaped group of humpback whales, as shown in Eq. (28)

(28) $$D\left( {i + 1} \right) = {W}^{\prime}{.}{e^{al}}{.}\cos \left( {2\pi l} \right) + {D^*}\left( i \right)$$

(29) $$D\left( {i + 1} \right) = \left\{ {\matrix{ {{D^*}\left( t \right) - P{.}W\quad \quad\quad\quad\quad if\ r < 0.5} \cr {{W}^{\prime}{.}{e^{al}}{.}\cos \left( {2\pi l} \right) + {D^*}\left( i \right)\quad if \ r \ge 0.5} \cr } } \right.$$

**Figure 3:**
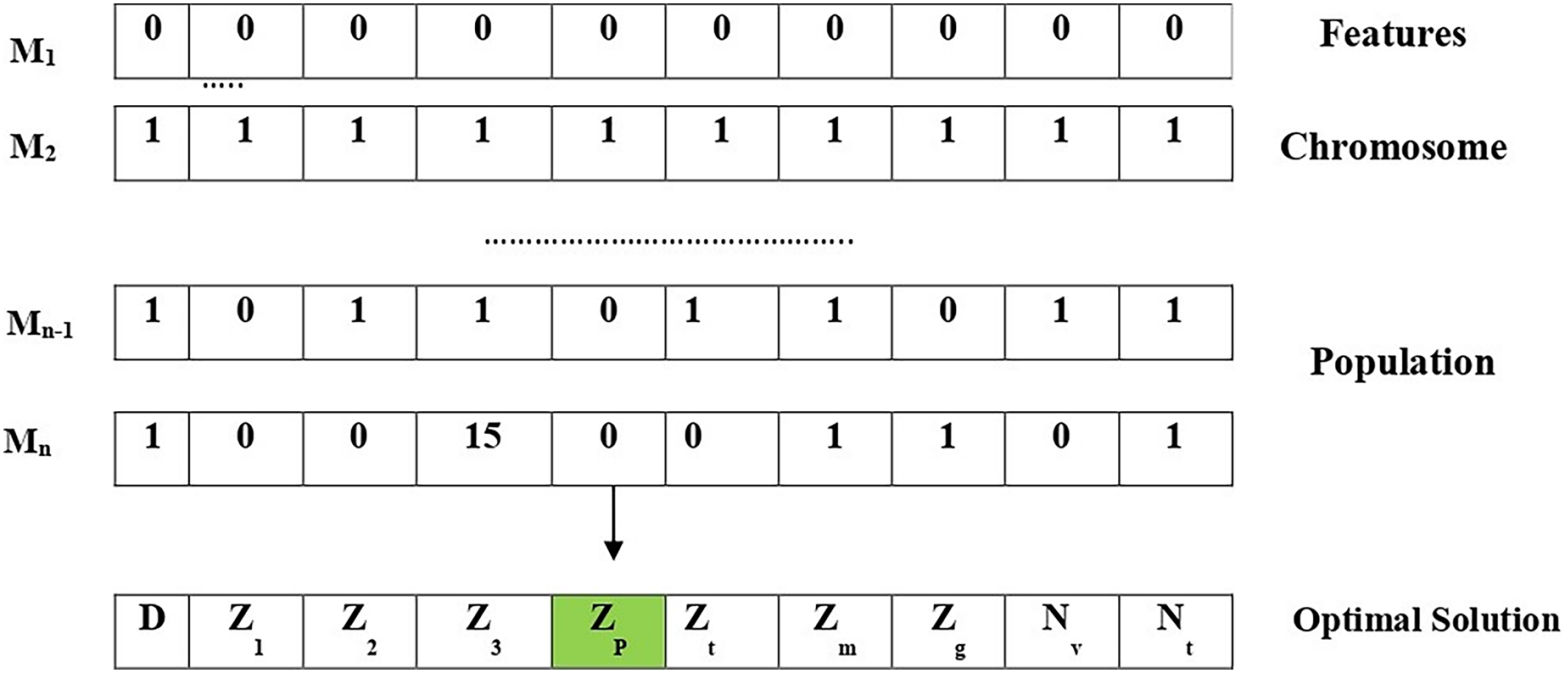
Solution representation of the proposed algorithm.

Equation (29) illustrates the statistical formula for updating the answer depending on the shrinking encircling technique or the spiral method, where $r$ is a random value between [0, 1]. $D$ may be used to look for the vector of the prey. To dissuade searchers from the reference whale, Vector $D$ has a random rate in the range [1, 1]. Its numerical expression is given as follows

(30) $$W = \left| {Q.{D_{randam}} - D} \right|$$

(31) $$D\left( {i + 1} \right) = {D_{randam}} - P{.}W$$

where ${D_{randam}}$ is the random position vector chosen among the available solutions and the solution representation of the proposed algorithm is shown in Fig. 3.

Initially, features within the chromosome should be chosen. As an outcome, the length of the chromosome is 10, pertaining to ten aspects that are depicted in Fig. 3. Considering the chromosome, each feature is assigned a unique value, *i.e*., one when selected or 0 otherwise. Following that, original chromosomes are chosen at random to form the population. After that, several search agents were selected for mating in order to produce offspring chromosomes based on the fitness value associated with each solution (*i.e*., chromosome). Later, a fitness function is employed for calculating the fitness value. The lower the fitness value, the better the solution. The “search agents” are then selected from the current population based on the fitness function. The nature of the ILW-LSTM lies in the hypothesis that mating two best solutions could generate an optimal solution.

#### Fitness function

The major contribution towards this work is to improve classification accuracy with a limited amount of calculation time. The fitness estimation of each solution is evaluated after initialization and saved for further use. The proposed ILW modifications to this approach are designed to reduce the error between the projected and actual LSTM output. The advantage of traditional WOA for evaluating unimodal functions is its improved coordination of exploitation. Additionally, it excels at exploring multimodal functions and improves convergence speed throughout an entire iteration. Along with its positive aspects, WOA also has certain disadvantages, such as the fact that it cannot handle all optimization issues and that it searches for the global optimum with slow convergence. The planned ILW is used to address these issues. In the traditional WOA, the variable $r\;$ was utilized, with a value chosen at random between [0, 1]. The $r$ value is dependent on a formulation involving two criteria to further increase the performance.

(32) $$r = \displaystyle{{f\left( {i - 1} \right) - f\left( i \right)} \over {f\left( {i - 1} \right)}}$$

(33) $$r = 0.9 \times {\raise0.7ex\hbox{${f\left( i \right)}$} \!\mathord{\left/ {\vphantom {{f\left( i \right)} {max\left( {f\left( i \right)} \right)}}}\right.} \!\lower0.7ex\hbox{${max\left( {f\left( i \right)} \right)}$}} + 0.1$$

Here, the terms $f\left( {i - 1} \right), f\left( i \right)$ denote the fitness value of the solution in the prior iteration, and the current iteration respectively. Additionally, $max\left( {f\left( i \right)} \right)$ denotes the maximum of all fitness values. The $r$ value for that answer is based on Eq. (32) if the fitness value of the current solution is greater than that of the prior solution. However, if the fitness value of the new solution is not greater than that of the previous solution and the value of $r$ depends on Eq. (33).

## Experiment

The experiment was implemented to evaluate the ILW-LSTM model for feature-level sentiment analysis on the Prompt Cloud dataset. The experimental design, data pre-processing, hyper-parameter settings, and performance measures were all described in depth in this section.

### Dataset description

Prompt Cloud generated this dataset, which has been combined into a usable dataset. It includes Amazon-generated phone reviews. The majority of reviewers have awarded unlocked mobile phones four-star and three-star ratings. To identify patterns in reviews, ratings, and price as well as the relationships between them, PromptCloud examined 400 k customer reviews of unlocked mobiles available on Amazon.com. Below is a list of the fields.

- Title of the product
- Trade mark
- Cost
- Ranking
- Review text
- How many consumers thought the review was beneficial

The data was attained in December 2016 by the flatterers used to provide our data extraction services. The reviews are around 230 characters long on average. It also revealed that longer reviews are frequently more beneficial and that there is a favourable relationship between price and rating.

### Hyper-parameters setting

The accuracy was optimized and the hyper-parameter was tuned using the improvised local search whale optimization approach. The hyper parameters measures in the suggested approach are shown in Table 1.

**Table 1:** Hyperparameters value in the proposed model.

Parameter	Values
LFMO	
Number of agents	20
Max iteration	500
Probability of mutation	0.2
ILW	
Number dimension	3
Upper bound	10.0
Lower bound	−10.0
Number of particles	50
Max iteration	100
Spiral coefficient	1

### Experimental setup

To create deep learning models, a variety of tools and libraries are available. Keras is the preferred tool. Tensor Flow was used as Keras’ backend since it was GPU-compatible. The following computer specs were used for deep learning experiments: The Python program is used to execute the experiment on a computer with 12 GB of RAM and an Intel TM core (7M) i3-6100CPU running at 3.70 GHz. The model was built and trained using Python programming. The system’s performance is shown by comparing its evaluation metrics to those of other systems currently in use.

### Evaluation metrics

Similar studies will compare the classification achievements from sentiment classification with the confusion matrix measurements to demonstrate the accuracy of the methodology. The confusion matrix is employed to determine measurement values for accuracy, precision, recall, and F1-score. One class must always be designated as positive and the other as negative when dealing with two-class categorization issues. Take into account the test set, which consists of both positive and negative samples. The goal of every classifier is to assign a class to each sample; however, certain classifications might not be acceptable. The number of ‘true positive’, ‘true negative’, ‘false positive’, and ‘false negative’ samples are counted to evaluate the performance of the classifier.

- ***True Positive***: Number of examples when both the correct class label and the anticipated class label are positive.
- ***True Negative***: The number of instances where the actual class label is right while the projected class label is false.
- ***False Positive***: Number of cases where the actual class label is inaccurate while the projected class label is positive.
- ***False Negative***: Number of cases where the actual class label is inaccurate while the projected class label is negative.

Our suggested ILW-LSTM model is put to the test using standard performance evaluation. The following metrics can be used to evaluate the model:

- **Accuracy:** It may be identified as the percentage of all accurately classified occurrences in the overall count of instances.

${Accuracy} = {\; }\left( {\displaystyle{{\left( {{Tp} + {Tn}} \right)} \over {\left( {{Tp} + {Tn} + {Fp} + {Fn}} \right)}}} \right)$

- **Precision:** It is characterized by the proportion of correctly classified positive occurrences to the total number of positively anticipated instances.

${Precision} = {\; }\left( {\displaystyle{{{Tp}} \over {\left( {{Tp} + {Fp}} \right)}}} \right)$

- **Recall:** It is described as the fraction of suitably categorized positive instances from the total count of positive instances.

${Recall} = {\; }\left( {\displaystyle{{{Tp}} \over {\left( {{Tp} + {Fn}} \right)}}} \right)$

- **F1-score:** It can be defined as the average harmonic between precision and recall.

${F}{1_{{Score}}} = {\; }\left( {\displaystyle{{2{\; }\left( {{Recall\; } \times {precision}} \right)} \over {{Recall} + {precision\; }}}} \right)$

### Experimental results

**Figure 4:**
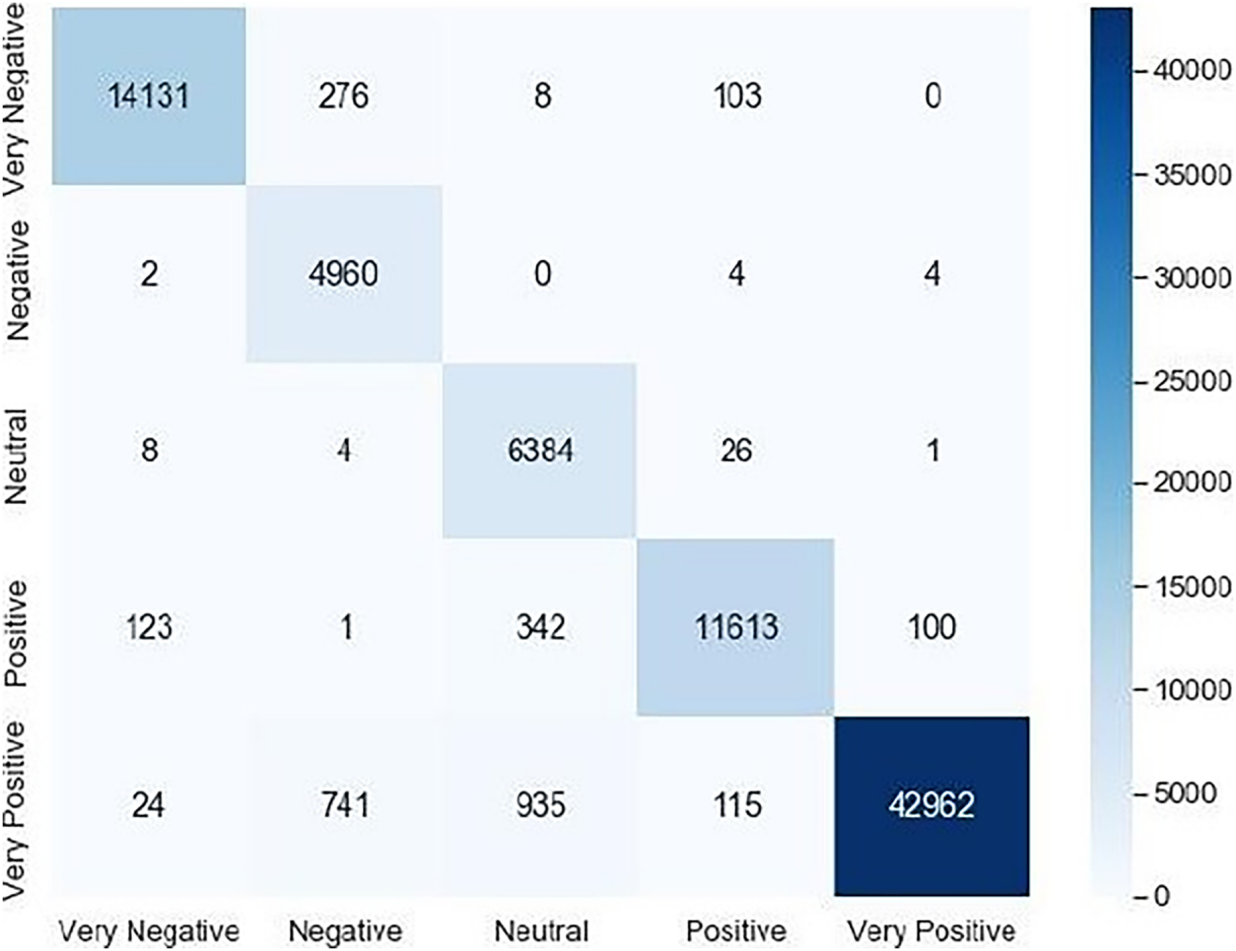
Confusion matrix.

**Figure 5:**
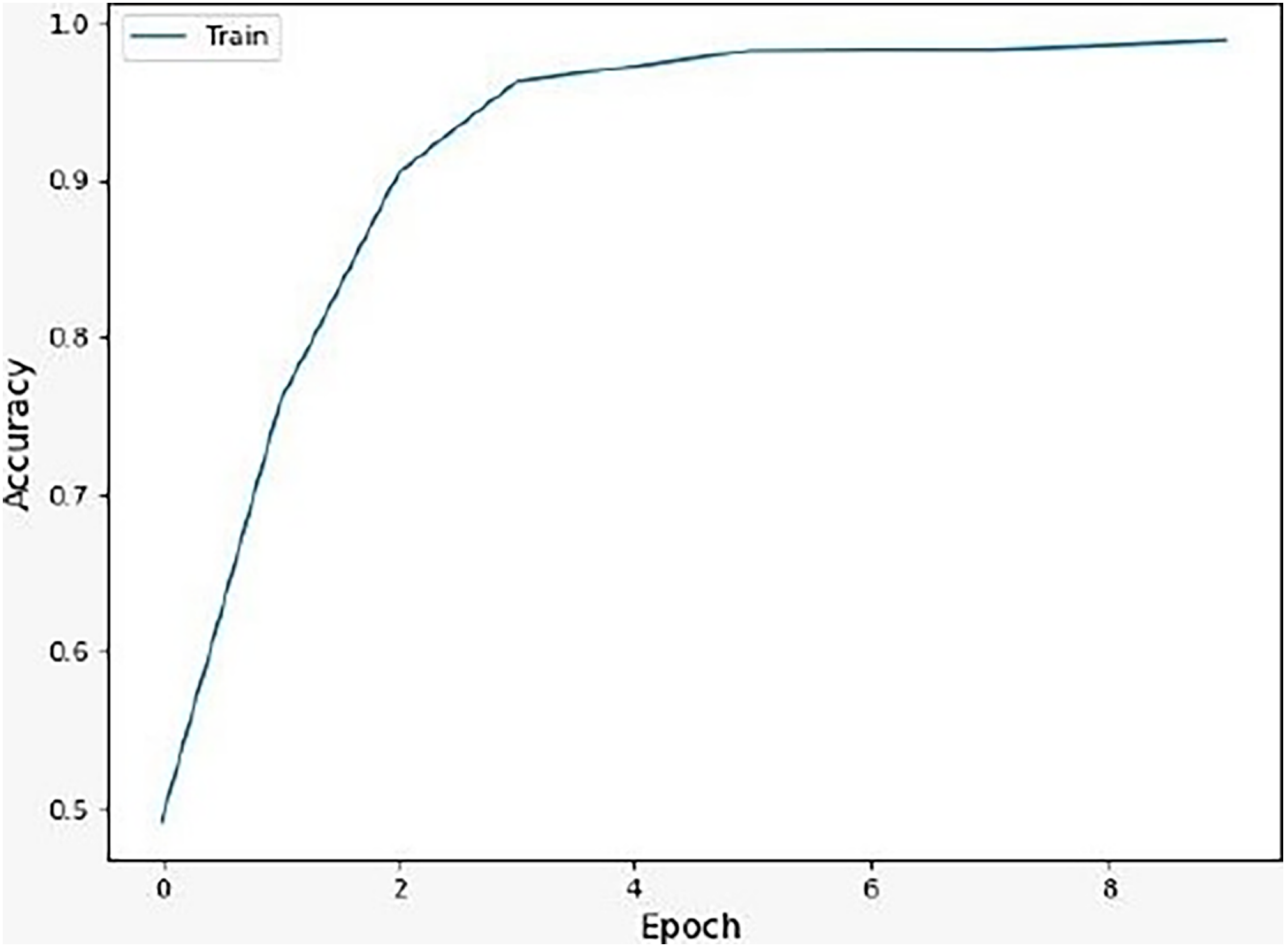
Accuracy of the suggested model.

Accuracy, recall, precision and F1-score are calculated according to the obtained confusion matrix that is given in Fig. 4:

We noticed that the suggested hybrid model’s classification results can be distinct throughout the testing of the proposed models. In other words, the identical input was categorized as negative by the suggested model. The accuracy of the training and validation datasets compared to the training epochs is shown in Fig. 5. The values for the epoch are on the x-axis, while the values of accuracy are on the y-axis. The accuracy of the proposed model was found to be 97 percent. To assess the false positive and true positive of our methods, the authors like to draw the receiver operating characteristic (ROC) curves for our systems in the additional Fig. 6.

**Figure 6:**
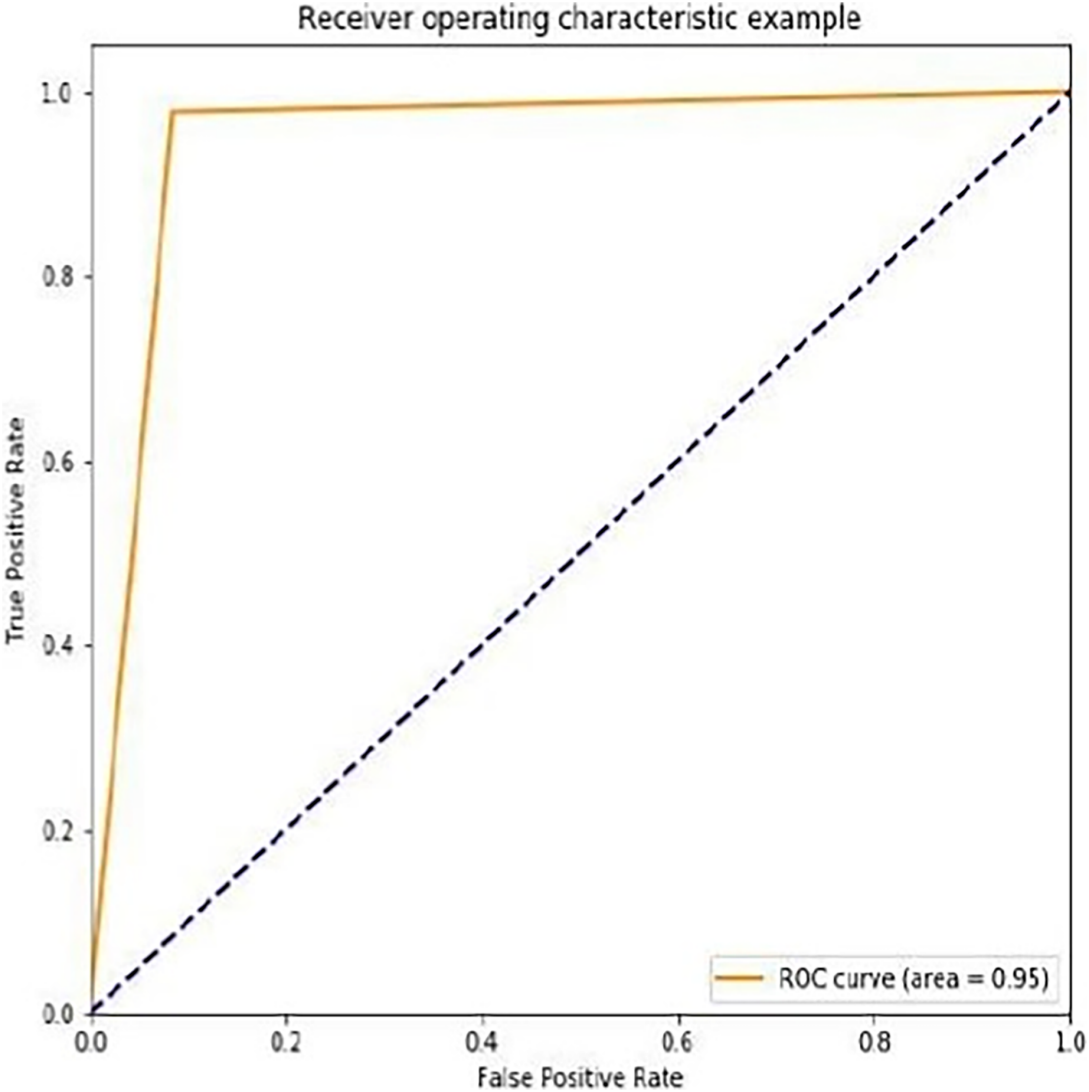
ROC curve for the proposed methodology.

The ROC curve for the suggested method is displayed in Fig. 6. The ROC curves demonstrate how well our model performs at various 0–1 thresholds. Based on the ROC for the ILW-LSTM approach, this work discovered that the suggested technique has good effectiveness for feature-level sentiment analysis.

**Figure 7:**
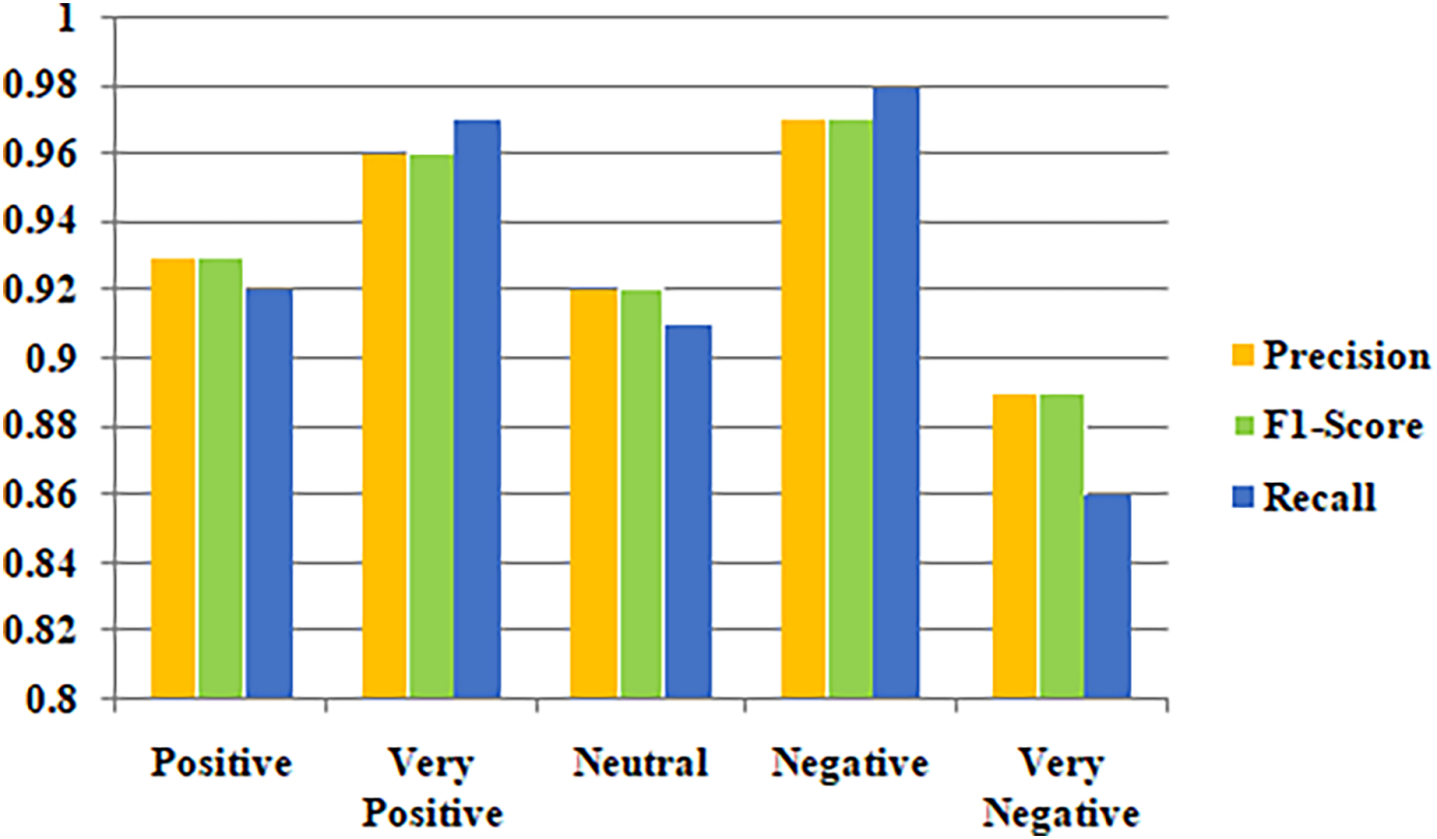
Classification outcomes based on precision, F1-score and recall.

Metrics for class-based categorization for the suggested models are shown in Table 2. Aside from accuracy, measures like F1-score, recall, and precision were also considered for evaluating the effectiveness of the model because the dataset utilized in the research is imbalanced. The suggested models performed best in terms of class-based F1-score, precision, and recall score for the negative class. Figure 7 illustrates the precision, F1-score and Recall values for the classification outcome.

**Table 2:** Classification outcome.

Class	Positive	Very positive	Neutral	Negative	Very negative
Metrics					
Precision	0.93	0.96	0.92	0.97	0.89
F1-score	0.93	0.96	0.92	0.97	0.89
Recall	0.92	0.97	0.91	0.98	0.86

### Comparative analysis

To demonstrate the efficacy of the proposed model’s performance, the findings from the suggested work are compared to those from several other current methodologies. These findings clearly proved the value of the ILW-LSTM technique and encouraged several research projects to create a sentiment categorization system based on deep learning algorithms. As a consequence, the findings of the suggested model have been compared with those of current methods in this section.

**Figure 8:**
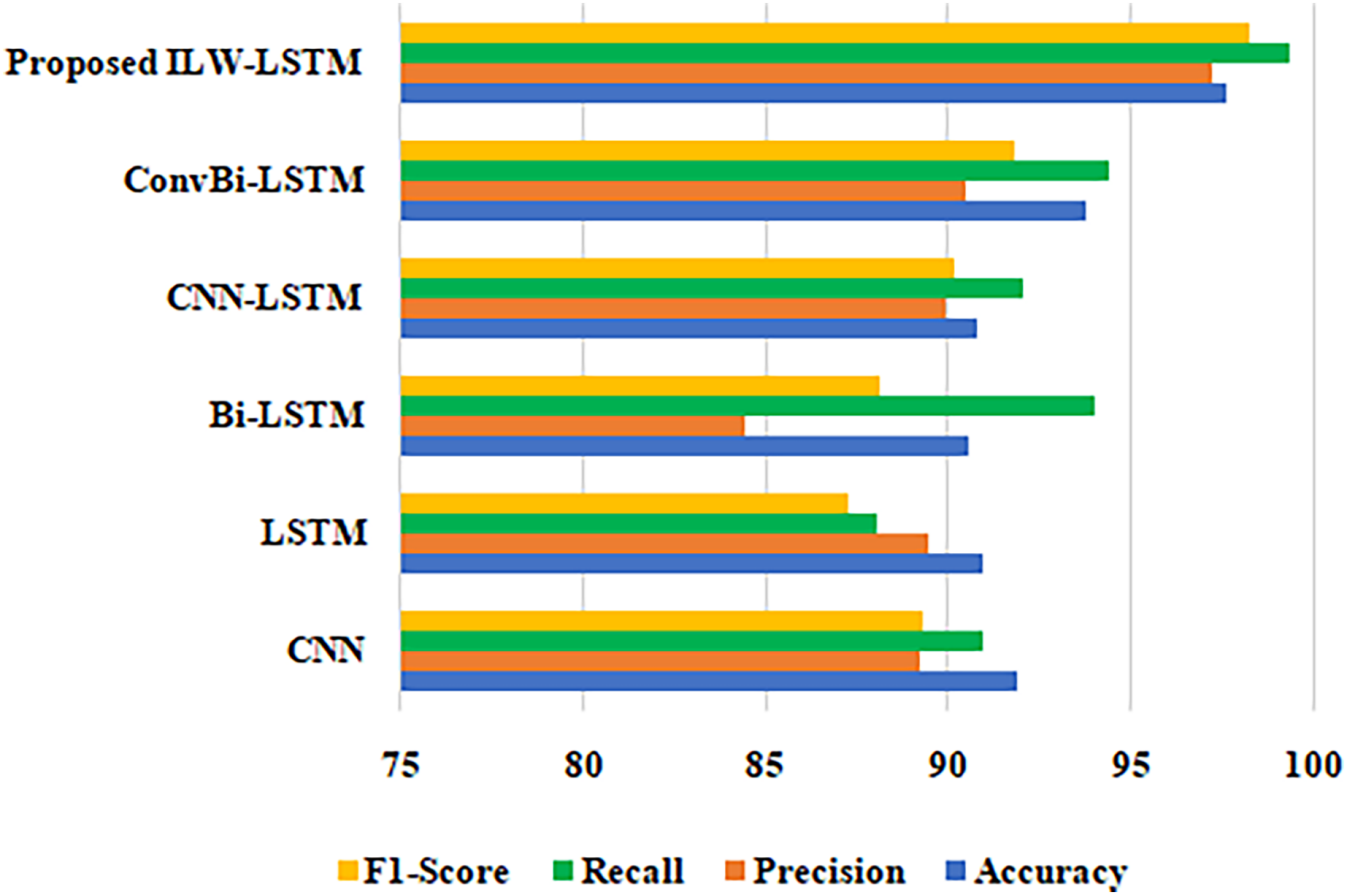
Comparison of different approaches in terms of F1-score, recall, precision and accuracy.

A comparison of the different methods using the suggested methodology is shown in Table 3 for clarity. Accuracy, precision, recall, and F1-score are the factors used in the comparison analysis. In this case, the suggested methodology values for accuracy, precision, recall, and F1-score are 97.61%, 97.24%, 99.35%, and 98.27%, respectively, when compared to those of CNN, LSTM, Bi-LSTM, CNN-LSTM, and ConvBi-LSTM. On PromptCloud datasets, the ILW-LSTM model performed far better than other deep learning models. Figure 8 shows a comparison of the accuracy, precision, F1-score and recall of several methodologies.

**Table 3:** Overall performance analysis of proposed and existing methods.

Performance metrics	Accuracy	Precision	Recall	F1-score
Techniques				
CNN	91.89	89.19	90.92	89.26
LSTM	90.94	89.48	88.06	87.86
Bi-LSTM	90.57	84.42	93.99	88.1
CNN-LSTM	90.81	89.96	92.02	90.13
ConvBi-LSTM	93.76	90.5	94.42	91.81
Proposed ILW-LSTM	97.61	97.24	99.35	98.27

## Conclusion

Multi-class sentiment analysis has consistently been a complex problem that has piqued the interest of academics owing to its broad range of applications. In this study, the ILW-LSTM is proposed to determine the polarity of consumer reviews. The PromptCloud dataset is utilized in the experiments. The suggested model begins with the data pre-processing stage to transform raw data into an understandable format. The pre-processed data then go through a feature extraction procedure using a modified inverse class frequency algorithm based on Log term frequency. The mayfly method was chosen in this instance for feature selection due to its excellent exploration capacity, and by leveraging levy flight as a reliable hybrid strategy, it also obtains greater exploitation capacity. Finally, the selected data is transformed to the ILW-LSTM, which categorizes the sentiment of the customer reviews as ‘positive’, ‘negative’, ‘very positive’, ‘very negative’, and ‘neutral’. Focused on evaluation metrics like precision, recall, f-score, and accuracy, the proposed ILW-LSTM approach is compared to the existing CNN, LSTM, CNN-LSTM, Bi-LSTM and ConvBi-LSTM techniques. The outcome shows that, when compared to existing sentiment classifiers, the ILW-LSTM achieves the greatest level of performance for the datasets. The ILW-LSTM classifier used in the sentiment analysis achieves a classification accuracy of about 97% which is more effective than existing techniques.

As far as future scope, there is still scope for multiclass sentiment classification in the present study. The proposed method could be strengthened using a novel hybrid optimization technique. For the systems under study, additional feature selection technique and to incorporate a data set comprising of emojis may be considered to further improve the classification performance.

## Supplemental Information

10.7717/peerj-cs.1336/supp-1 Supplemental Information 1 Code for executing Fine Grained Sentiment Analysis.

## Additional Information and Declarations

### Data Availability

The following information was supplied regarding data availability: The code is available in the Supplemental File.
